# 4530 Elucidating the Influence of Chemotherapy (melphalan) and /or C. difficile toxin B Exposure on Beta-catenin Protein Expression in Caco-2 Monolayers

**DOI:** 10.1017/cts.2020.297

**Published:** 2020-07-29

**Authors:** Jailene Canales, Alison Weiss, Senu Apewokin

**Affiliations:** 1 UC Physicians

## Abstract

OBJECTIVES/GOALS: We previously reported that genetic polymorphisms in the beta-catenin gene (CTNNB) are associated with the development of *Clostridiodes difficile* colitis during autologous stem cell transplantation (https://www-ncbi-nlm-nih-gov.proxy.libraries.uc.edu/pubmed/29594489). To biological validate these findings, we sought to evaluate the development of chemotherapy-associated *Clostridiodes difficile* infections by assessing the effect of C.difficile toxin B (TcdB) and of using melphalan in beta-catenin protein expression in Caco2 cells. METHODS/STUDY POPULATION: To determine the effect of melphalan and/or C.difficile toxin B on expression of *Beta-catenin* from human gut epithelial cells:

- Adenocarcinoma cells (Caco-2) cells were seeded and allowed to grow into monolayers
- Monolayers were treated with PBS, TcdB, melphalan and/or TcdB + melphalan for 24 hours and then washed with PBS
- Immunofluorescence was measured on the monolayers to visualize three markers -DAPI-Nuclear Stain (blue),Actin-ccytoskeletal stain (red), B-Catenin (green)
- Analysis of images with ImageJ (NIH). Statistical analysis of the effect of TcdB and/or melphalan on β-catenin protein levels was determined by One-way ANOVA

Cells stained with a primary anti-β catenin antibody and an Alexa-488 secondary antibody were evaluated by flow cytometry to quantify the effect of melphalan and/or C. difficile toxin B on Caco2 cells. RESULTS/ANTICIPATED RESULTS: Immunofluorescent intensity was higher in the control (PSS exposed) cells when compared to melphalan, TcdB and mephalan+TcdB exposed cells (p = 0.026, 0.004 and 0.049 respectively) DISCUSSION/SIGNIFICANCE OF IMPACT: A significant difference was seen in β catenin expression in Caco-2 monolayers exposed to TcdB and/or melphalan. These data support the a role of β-catenin in the pathophysiology of CDI during chemotherapy and support GWAS findings reporting a difference in CDI susceptibility based on β-catenin genotype.

